# Evolutionary Landscape of Tea Circular RNAs and Its Contribution to Chilling Tolerance of Tea Plant

**DOI:** 10.3390/ijms24021478

**Published:** 2023-01-12

**Authors:** Jin Huang, Yanli Wang, Jie Yu, Fangdong Li, Lianghui Yi, Yunze Li, Na Xie, Qiong Wu, Lidiia Samarina, Wei Tong, Enhua Xia

**Affiliations:** 1 State Key Laboratory of Tea Plant Biology and Utilization, Anhui Agricultural University, Hefei 230036, China; 2 Institute of Sericultural Research, Anhui Academy of Agricultural Sciences, Hefei 230031, China; 3 Tea Research Institute, Anhui Academy of Agricultural Sciences, Hefei 230031, China; 4 Federal Research Centre the Subtropical Scientific Centre, The Russian Academy of Sciences, Sochi 354002, Russia

**Keywords:** *Camellia sinensis*, circular RNA, evolutionary landscape, cold stress, noncoding RNAs

## Abstract

Chilling stress threatens the yield and distribution pattern of global crops, including the tea plant (*Camellia sinensis*), one of the most important cash crops around the world. Circular RNA (circRNA) plays roles in regulating plant growth and biotic/abiotic stress responses. Understanding the evolutionary characteristics of circRNA and its feedbacks to chilling stress in the tea plant will help to elucidate the vital roles of circRNAs. In the current report, we systematically identified 2702 high-confidence circRNAs under chilling stress in the tea plant, and interestingly found that the generation of tea plant circRNAs was associated with the length of their flanking introns. Repetitive sequences annotation and DNA methylation analysis revealed that the longer flanking introns of circRNAs present more repetitive sequences and higher methylation levels, which suggested that repeat-elements-mediated DNA methylation might promote the circRNAs biogenesis in the tea plant. We further detected 250 differentially expressed circRNAs under chilling stress, which were functionally enriched in GO terms related to cold/stress responses. Constructing a circRNA-miRNA-mRNA interaction network discovered 139 differentially expressed circRNAs harboring potential miRNA binding sites, which further identified 14 circRNAs that might contribute to tea plant chilling responses. We further characterized a key circRNA, *CSS-circFAB1*, which was significantly induced under chilling stress. FISH and silencing experiments revealed that *CSS-circFAB1* was potentially involved in chilling tolerance of the tea plant. Our study emphasizes the importance of circRNA and its preliminary role against low-temperature stress, providing new insights for tea plant cold tolerance breeding.

## 1. Introduction

Chilling stress is one of the major environmental factors affecting the crop yield and distribution worldwide. Investigation of the regulation mechanism on how crops respond to chilling stress is of great significance to breed new varieties with high resistance to meet global needs. The tea plant is an important thermophilic cash crop that plays roles in economic development, especially in developing countries. The tea plant is rich in polyphenols such as catechins, theanine, and caffeine, which provide tea with good flavor and also benefit to people’s health [1]. This also makes tea one of the most widely consumed drinks in the world after water [2]. In recent years, low-temperature and extreme weather conditions have crucially restricted the crop growth and yield, quality, and geographic distribution [3,4], particularly for the tea plant, which is vulnerable to low temperature [5].

In the last several decades, great advances have been obtained in deciphering the molecular mechanism underlying low-temperature responses in the tea plant. Several studies have explored the gene expression patterns of the tea plant under chilling stress and discovered a large number of differentially expressed genes that might be associated with chilling tolerance [6,7,8,9,10]. Genes encoding transcription factors, such as AP2/ERF, bHLH, bZIP, MYB, and WRKY, have been proved to play vital roles in response to low-temperature stress [3,4,6,7,9,11]. The CBF transcription factors and the ICE1-CBF-COR regulatory pathway are critical and conserved for plant adaptation to low temperature. Several important cold-responsive genes, such as *bZIP18*, which negatively regulate the cold tolerance of the tea plant [12], and *UGT91Q2* [13], were cloned and verified, yet their regulating mechanism remains unclear. microRNAs were also demonstrated to be involved in the regulation of cold stress in tea plant. For example, the miR171 family of tea plants was significantly up-regulated in cold stress but with low expression in cold-susceptible cultivars, suggesting that the expression of miR171 may be associated with the cold sensitivity of different tea plants [14]. These abovementioned progresses presented novel observations, explaining how the tea plant responds to cold stress mostly in gene expression level. However, cold stress responses are a complicated process in plants, which involves lots of functional genes, transcription factors, epigenetic modifications, non-coding RNAs, and a series of complex regulatory networks among them [8,10,11,12,13,14].

Unlike miRNA and linear genes, circRNA is a novel type of covalently closed RNA molecule that is head-to-tail-joined through back-spliced junction sites, and is found to play potential roles in regulating plant growth as well as biotic and abiotic stress responses [15,16,17,18]. For example, circRNAs in kiwifruit have a specific expression response to pathogen invasion [19]. The expression levels of circRNAs varied when maize and *Arabidopsis* were subjected to different degrees of drought stress, indicating that circRNAs might be an effective molecular marker for plant drought response [20]. In the case of cold response, Zuo et al. observed that 102 circRNAs act as the sponges of miRNAs to mediate the cold response in tomato fruit [21]. Specifically, overexpression of the grape *Vv-circATS1*, a circRNA derived from grape glycerol-3-Pacyltransferase gene, improved the cold tolerance of transgenic *Arabidopsis* plants [16]. To investigate the function of circRNAs in tomato leaves’ response to low-temperature stress, Yang et al. identified 1759 low-temperature-induced circRNAs, and revealed that 383 differentially expressed circRNAs and their parental genes were functionally enriched in the regulation of metabolism, signal transduction, and environment adaptation pathways, revealing the vital roles of circRNA and its corresponding parental genes in response to low-temperature stress [22]. These studies demonstrated that circRNAs possess the abilities to respond to biotic and abiotic stresses by regulating the expression of stress-related genes. A recent study has also characterized the circRNAs in the leaf and bud of the tea plant and revealed that the tea plant circRNAs show tissue-specific expression. Meanwhile, it is also found that there is a potential interaction and regulation relationship between circRNA and mRNA or miRNA [17]; however, how circRNAs regulate the chilling response of tea plants has not been investigated. In addition, compared with the circRNAs identified in animals, the circRNAs from plants harbor several important characteristics that clearly distinguish them from circRNAs in animals; for example, the flanking intron sequence of plant circRNAs is shorter, and 20–50 nt flanking sequences can drive the occurrence of plant circRNAs [16]. Thus, understanding the evolutionary characteristics of circRNA in the tea plant and its feedbacks under chilling stress will help to elucidate the vital roles of circRNAs.

In the current report, we systemically identified and examined the circRNAs of the tea plant and their expression patterns under chilling stress. Through deep bioinformatics analysis and functional experiments, in conjunction with mRNA and miRNA analysis, we aimed to reveal the exhaustive characteristics, expression and evolutionary patterns, and the potential functional roles of tea plant circRNAs on chilling tolerance. The obtained results will not only provide novel insights into the evolution and expression dynamics of tea circRNAs, but will also highlight the important roles of circRNAs in regulating plant cold tolerance.

## 2. Results

### 2.1. Characterization and Validation of circRNAs in Tea Plants under Chilling Stress

**Figure 1:**
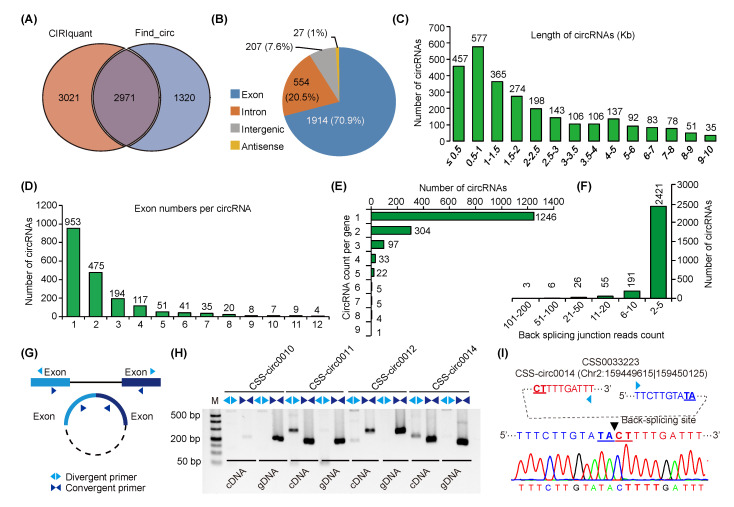
Characteristics and sequence validation of tea plant circRNAs. (**A**) Two state-of-the-art tools (find_circ and CIRIquant) were used for circRNAs identification in tea plant. (**B**) Four major types (exon, intron, intergenic, and antisense) of the identified circRNAs. (**C**) Length distribution of the circRNAs. (**D**) Number of exons harbored by the circRNAs. (**E**) CircRNA counts produced from parental genes. (**F**) Back-splicing junction reads count of circRNAs. (**G**) Design method of convergent and divergent primers. (**H**) PCR amplification of the random selected circRNAs for validation. (**I**) Sanger sequencing validation of the back-spliced sites of circRNA use CSS0033223 as an example.

To identify tea plant circRNAs under chilling stress, we performed chilling treatment of 15 tea plant samples at different time points and performed in-depth circular RNA sequencing. A total of 1521 million clean reads were generated in all samples and an average of 10.15 Gb of clean bases were obtained with a Q30 more than 97% (Table S1). Two state-of-art circRNAs identification tools, CIRIquant and Find_circ, were then used to characterize circRNAs as previously recommended. Totals of 5992 and 4291 unique circRNAs were predicted in CIRIquant and Find_circ, respectively, of which 2971 circRNAs derived by both tools were obtained (Figure 1A). Under expectation and as also illustrated in previous studies, the ratio of shared circRNAs harbored by the two tools (40% between the two tools), as well as among different treatment groups (413 and 216 shared by all samples in CIRIquant and Find_circ, respectively), was quite low (Figure S1A,B). After removal of 269 circRNAs with too long a sequence length (>10 kb), we finally obtained 2702 high-confidence circRNAs (Figure 1A and Table S2). According to the gene annotation, we found four types of circRNAs in the tea plant, including exonic circRNAs (1914, 70.9%), intronic circRNAs (554, 20.5%), intergenic circRNAs (207, 7.6%), and antisense circRNAs (27, 1%) (Figure 1B, Table S2). Most of the identified circRNAs were less than 2000 bp in length, and more than 70% (1914) of the identified circRNAs contained 1–4 parental gene-derived-exons (Figure 1C,D, Table S2). Chromosomal distribution analysis illustrated that circRNAs are widely located in the tea plant chromosomes (Figure S1C). We then annotated the parental genes of circRNAs and identified a total of 1717 unique protein-coding genes of all the circRNAs, indicating that more than one circRNAs were generated of some parental genes, although most parental genes produced only one circRNA (Figure 1E, Table S3). Our analysis also showed that back-spliced sites of circRNAs were mostly supported by two unique reads (Figure 1F and Table S2). These results are helpful for us to further identify the characteristics of circRNAs in tea plants as well as in other plants.

PCR amplification and Sanger sequencing were applied to validate the circRNAs. Convergent and divergent primers were designed to amplify the linear fragments from cDNA or gDNA and the back-spliced junction sites in cDNA (Figure 1G,H, Table S4). As shown in Figure 1G, the divergent primers in gDNA failed to amplify the sequence around the back-spliced junction site, while both convergent and divergent primers could amplify the circulated sequence in cDNA. Sixteen of the 25 randomly selected circRNAs can be successfully validated in our experiments (Figure 1H and Figure S2A). The head-to-tail junction sequences were further confirmed by Sanger sequencing using divergent primers in the cDNA samples (Figure 1I and Figure S2B). Additionally, we also experimentally validated the expressions of these circRNAs by quantitative real-time PCR (qRT-PCR) and found that the circRNAs respond to chilling stresses in the tea plant (Figure S3).

### 2.2. Conservation Assessment of circRNAs between Tea Plant and Other Plant Species 

**Figure 2:**
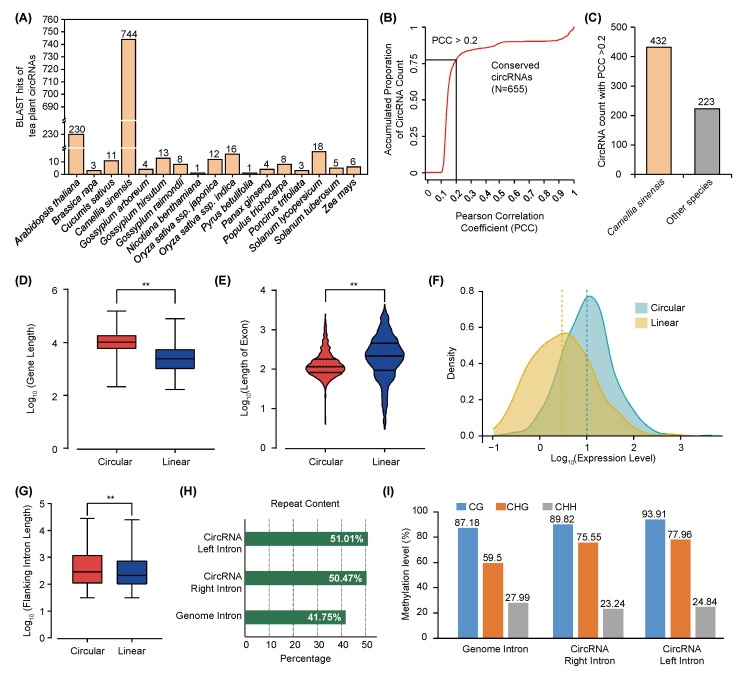
Conservation assessment of circRNAs in diverse plant species. (**A**) Similarity analysis of tea plant circRNAs with other plant species using junction sequences. (**B**,**C**) Similarity analysis of tea plant circRNAs with other plant species using *K-mer*-based method. (**D**) Comparison of gene lengths between genes with and without detectable circRNAs. (**E**) Exons length between genes with and without detectable circRNAs. (**F**) Expression levels (FPKM) between genes that can produce circRNAs with genes without detected circRNAs. Linear and circular represent randomly selected genes without detectable circRNAs and genes with detectable circRNAs in tea plant genome annotation, respectively. (**G**) Flanking-intron length between linear transcripts and circRNAs. *Y*-axis in the above subfigures is indicated by log_10_ of gene length, exon length, and intron length (bp). ** indicates significant difference between groups ≤ 0.01. (**H**) Repeat sequence content of flanking intron regions of circRNAs and randomly selected genes. (**I**) Methylation patterns around flanking introns of circRNAs. Weighted methylation level was calculated here.

CircRNAs are highly abundant in eukaryotes, and some of them are thought to be evolutionarily conserved among plant species [15,23,24]. To test this, we conducted the conservatism of circRNAs of tea plants and 20 other representative plants from the PlantcircBase database (Figure S4A). By alignment of the junction sequences, we found 755 unique tea plant circRNAs whose junction sites were covered by the aligned region with other circRNAs from 17 plant species. However, 715 of them show conservation with a previous circRNA dataset characterized from *Camellia sinensis* leaf and bud. It showed that few identified circRNAs (<2%) in the tea plant were conserved in sequences with other plant species (Figure 2A and Table S5). Linear sequence comparison of circRNAs found that 1279 tea plant circRNA sequences show similarities with 3177 circRNA sequences from 15 plant species (Table S6). To further understand the conservation of circRNAs, we also evaluated the similarity between the tea plant and other species according to a new algorithm that employ the abundance of *K-mer* short motifs for noncoding RNAs. We found that 655 circRNAs (24.2% of total identified circRNAs) show *K-mer* similarity with the circRNAs in other species with a Pearson correlation coefficient greater than 0.2 (Figure 2B,C). Among those circRNAs, 432 were circRNAs from a previous identification of the tea plant, and only 8.3% of the identified circRNAs show conservation with circRNAs from other plant species (Figure 2C), indicating that the similarity of plant circRNAs is low in current circRNA datasets.

### 2.3. Circularization of Tea Plant circRNAs Is Affected by TE-Mediated DNA Methylation in Flanking Introns

We further compared the characteristics of circRNA-parental genes and the common protein-coding genes of the tea plant. We found that circRNA parental genes showed significantly longer gene length than the randomly selected linear genes without detectable circRNAs (Figure 2D). However, the exon length of circRNA parental genes is significantly shorter than the randomly selected linear genes not produced (Figure 2E). We also observed that genes without harbored circRNAs show a lower overall expression abundance compared to the genes producing circRNAs (Figure 2F). Previous studies have reported that the flanking introns and repeat sequences play vital roles in circRNAs biosynthesis [25,26,27,28]. We then calculated the flanking intron length of circRNAs in the tea plants and found that the length of flanking introns is significantly longer than that of randomly selected linear genes (Figure 2G). When we assessed the ratio of repetitive sequences in flanking introns, we observed that the repeat sequence content of circRNAs flanking introns is higher than that of the random intron regions of genome (Figure 2H). Further classification of the repeat sequences showed that long terminal repeats were the most abundant type in the flanking introns of circRNAs, accounting for 46.86%, 46.14%, and 37.7% of the total repetitive sequences in left and right introns, and randomly selected genome introns, respectively (Figure S4B). DNA methylation level analysis revealed that the flanking intron regions of the circRNAs were more heavily methylated compared to the methylation levels in genomic intron regions (Figure 2I), implying that the hypermethylation of the flanking introns of tea plant circRNAs may mediate its circRNAs formation.

### 2.4. Expression Response of Tea Plant circRNAs to Chilling Stress

**Figure 3:**
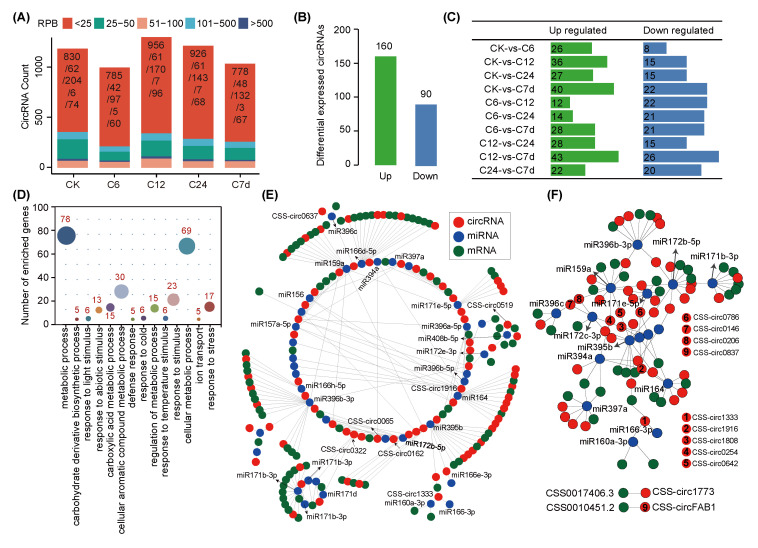
Differential expression and interaction network analysis of circRNAs in tea plant. (**A**) The expression pattern of circRNAs using RPB (junction reads per billion clean reads). (**B**) Differentially expressed circRNAs identified under chilling stress in tea plant. (**C**) Up- and down-regulated circRNAs identified among different comparisons. (**D**) Functional annotation of differentially expressed circRNAs. (**E**) circRNA-miRNA-mRNA interaction network of tea plant under chilling stress. (**F**) The key circRNAs potentially interacting with well-known cold-related miRNAs in the network are highlighted.

We calculated the expression level of circRNAs using junction reads per billion clean reads (RPB) and assessed the distribution of circRNA expressions. The results showed that the expression abundance of circRNAs differed between chilling treatment with most circRNAs being low-expressed (<100 RPB) and a small number being high-expressing (500–1500 RPB) (Figure 3A, Table S7). We then identified the differentially expressed circRNAs (DECs) response to chilling stress in different treatment timelines of the tea plant (Table S8). Ultimately, we obtained 250 differentially expressed circRNAs, of which 160 were up-regulated (Log_2_FC > 1) and 90 were down-regulated (Log_2_FC < −1) (Figure 3B,C, Table S9). We also characterized 6384 differentially expressed genes under chilling treatments, of which 4321 and 2513 genes were up and down-regulated, respectively (Figure S5A), which indicated that more genes were up-regulated to participate in the chilling tolerance process, thus improving the resistance of the tea plant. We performed gene ontology (GO) enrichment analyses on the parental genes of circRNAs, which were differentially expressed under chilling stress, and found that those genes were mainly enriched in secondary metabolic processes, abiotic stress responses, cold stress responses, defense responses, and ion binding function categories (Figure 3D and Table S10). Therefore, these differentially expressed circRNAs may play an important role in the tea plant response to chilling stress.

### 2.5. Tea Plant circRNAs Function as miRNAs Sponges during Chilling Stress

In order to deeply resolve the interaction between circRNAs and miRNAs, we performed high-throughput sequencing of small RNA in the tea plant under chilling stress. Results demonstrated that the length of small RNAs was mainly concentrated between 21 nt and 24 nt (Figure S5B). Finally, 129 conserved miRNAs were obtained by comparing and analyzing them with known plant miRNAs in the miRBase database (Figure S5C,D, Table S11). The number is comparable to those identified in cotton [29], radish [30], and pitaya [31], but much larger than those of melon [32] and mulberry [33]. We then predicted the candidate circRNAs targeted by miRNA, and constructed the circRNA-miRNA-mRNA network (Figure 3E,F). We found that, of all the 250 differentially expressed circRNAs under cold stress, one hundred and thirty-nine of them were predicted to serve as the putative sponges of 116 miRNAs, and then to regulate 679 differentially expressed mRNAs (Tables S12 and S13). Remarkably, under chilling stress, the differentially expressed circRNAs could target multiple well-known miRNAs related to the response to chilling stress, such as miR166, miR172, miR396, and miR408 (Figure 3E,F). Target genes of these miRNAs were enriched in GO terms such as response to stress, response to cold, and response to abiotic stimulus and glycosyltransferase activity (Figure S5E, Tables S14 and S15).

### 2.6. CSS-circFAB1 Contributes to Tea Plant Cold Tolerance

**Figure 4:**
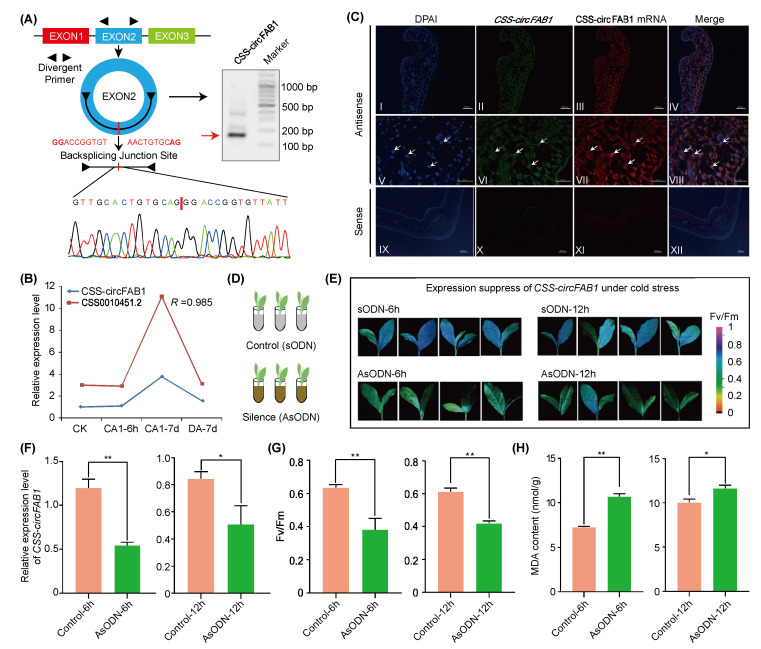
Circular RNA *CSS-circFAB1* contributes to tea plant cold tolerance. (**A**) Validation of *CSS-circFAB1* sequences by PCR and Sanger sequencing. (**B**) Relative expression levels of *CSS-circFAB1* and CSS0010451.2 genes under cold acclimation revealed by qRT-PCR and their expression correlation. (**C**) FISH experiment of *CSS-circFAB1* in tea plant leaves (I, II, V, and VI). The FISH of *CSS-circFAB1* mRNAs is shown for comparison (III, VII). IX-XII: Control sense probes. Scale bars = 100 μm (I–IV and IX–XII); 50 μm (V–VIII). (**D**) A schematic diagram of antisense oligodeoxynucleotides-based gene silencing of *CSS-circFAB1*. (**E**) Determination of the net photosynthetic rate and the maximum photochemical efficiency of photosystem II (*Fv/Fm*) of *CSS-circFAB1* silenced (6 h and 12 h) and control tea plants. (**F**) Relative expression levels of *CSS-circFAB1* feeding with AsODN at 6 h and 12 h compared to control. (**G**) *Fv*/*Fm* values in *CSS-circFAB1* silenced and control tea plants. (**H**) MDA content of *CSS-circFAB1* silenced samples compared to control. * *p* value < 0.05; ** *p* value < 0.01.

During the above experiments, we found that a differentially expressed circRNA, CSS-circ0837, and its parental gene (CSS0010451.2) were up-regulated under chilling and cold acclimation (Figure S6A,B). Alignment analysis found that CSS0010451.2 was homologous to the *Arabidopsis FAB1* gene (AT1G74960), involved in fatty acid biosynthesis. This circRNA was then named as *CSS-circFAB1*. We experimentally verified the back-spliced junction sequence of *CSS-circFAB1* (Figure 4A). qRT-PCR experiments showed that both *CSS-circFAB1* and CSS0010451.2 were significantly up-regulated under cold acclimation with a positive correlation coefficient of 0.985 between each other (Figure 4B). The fluorescence in situ hybridization (FISH) experiment revealed that *CSS-circFAB1* mainly localizes in the cytoplasm (Figure 4C). However, CSS0010451.2 is localized exclusively in the cytosol. To further explore the function of *CSS-circFAB*1 under chilling stress, we adopted antisense oligonucleotides (AsODN) solution to suppress the expression of *CSS-circFAB1* (Figure 4D). After feeding with AsODN, the expression levels of *CSS-circFAB1*, as well as CSS0010451.2, decreased significantly at 6 h and 12 h (Figure 4F and Figure S6C,D). The control and treatment samples were then exposed to cold stress (0 °C) for 1 h. We found that severe cold damage with a significantly low *Fv/Fm* value and higher malondialdehyde (MDA) accumulation were observed in *CSS-circFAB1*-silenced tea plant seedlings (Figure 4E,G,H). These combined results indicate that *CSS-circFAB1* might positively regulate the cold tolerance of tea plant despite more evidences are needed.

## 3. Discussion

Circular RNAs are covalently closed single-stranded non-coding RNAs that are widespread in eukaryotic species [34]. Many studies have revealed that circRNAs have various functional roles, including regulating gene expressions, and binding miRNAs or proteins, involved in individual growth and development, as well as many other biological processes [17,23,27,35]. However, it is still unclear how circRNA evolves and evolutionary characteristics of circRNAs and how circRNA regulates the chilling tolerance in tea plants. In this study, we systemically identified the circRNAs of tea plant and characterized their expression patterns under chilling stress. In total, 2702 high-confidence circRNAs were identified. PCR amplification and Sanger sequencing further revealed that sixteen of the 25 randomly selected circRNAs were successfully validated, indicating an accuracy of 64% of circRNA identification in the present study. The accuracy is comparable to those found in many flowering plants (average of 56%) [17,24]. It should be noticed that while the accuracy of circRNA identification is acceptable in the study, its bioinformatics algorithm still needs further improvement [16,18,20]. Consistent with the results in previous findings in rice, *Arabidopsis*, wheat, and grape [16,24,36], we also found that exonic circRNAs are the most abundant circRNA type, which also indicate that circRNAs are conserved in plants and mainly generated from the coding regions. We also confirmed that the number and expression abundances of circRNAs identified varied between treatment time points, indicating that specific circRNAs presences and/or absences are often spatiotemporal in tea plants. Comparison of the circRNAs of tea plant with other plant species suggests that tea circRNAs shared a low sequence conservation (less than 10%) with other plant species. A latest study on plant circRNA evolution proposed that circRNAs in plants might originate recently in the evolutionary scale [15]. They also found that circRNAs are less conserved than other non-coding RNAs, such as miRNAs. In rice, most circRNAs are derived from young parental genes and are more conserved at the genus level (49.1% within *Oryza*) than in other dicotyledonous plant species using a plant multiple conservation score method [15]. These results showed a similar pattern of the conservation between circRNAs among plant species with the evidence we obtained in current report.

Our knowledge about circRNAs biogenesis in plants is still on the way. We characterized the flanking introns of circRNAs in the tea plant, and notably found that the length of flanking introns of circRNAs is significantly longer than that of randomly selected linear protein coding genes. This suggests that the formation of circRNAs is probably associated with the length of flanking introns, and reinforces the previous findings showing that flanking introns were critical for exon circularization [16]. It has also been reported that the repetitive sequences within circRNAs are important for circRNAs formation in animals [26,28]. This is further evidenced by the findings in the present study that a larger number of repetitive sequences were identified in the flanking intron regions of circRNAs. Among the repetitive sequences identified, LTR transposable elements (TEs) were the most enriched sequence types in the flanking intron region of circRNAs, consistent with the previous observations in the *Moso bamboo* genome [37]. Seldom reverse complementary sequences in flanking introns of circRNAs were found in the tea plant, which differs from the observations in animals [20,26,38], but agrees with the results in plants such as rice and *Arabidopsis* [24,39], cotton [40], and grape [16], indicating that the reverse complement of the flanking sequence may not be a common factor regulating circRNA formation in plants. Although it is well accepted that circRNAs originate from back-splicing, it is not clear how TEs stimulate circRNA production. We analyzed the DNA methylation levels in the upstream and downstream flanking intron regions of circRNAs, and found that the flanking intron regions of the circRNAs were highly methylated. This suggested that hypermethylation of the flanking introns of tea plant circRNAs may mediate the circRNAs formation, which is consistent with the findings in *Moso bamboo* [37], in which flanking introns of circularized exons are in a hypermethylated state. There might be two potential reasons that explain the correlation between the methylation level of the flanking sequence and circRNA generation. One is that the high methylation level in flanking introns could lower the elongation rate of RNA polymerase II and regulate the back-splicing of circRNAs; the other is that the competing between splicing factors and epigenetic factors affect the formation of circRNAs [37].

Increasing evidence in different studies has shown that circRNA responds to a variety of abiotic stresses, including temperature, drought, osmotic stress, and nutrient deficiency [16,20,21,35,41]. Plant circRNAs can act as sponges for miRNAs to regulate the expression of downstream target genes by competing with other endogenous RNAs [21]. Our study identified 250 differentially expressed circRNAs in tea plants under chilling stress; their functions were involved in the synthesis of secondary metabolites, stress response, and cold response, as also found in horticulture plants such as tomato and grape [16,21]. The differentially expressed circRNAs of tea plants were found to interact with many well-known miRNAs (e.g., miR171, miR395, miR396, and miR408) involved in the cold response [14,42]. This further supported the previous hypothesis that circRNAs may interact with miRNAs and act as its sponges to participate in the stress response (e.g., cold). Furthermore, we particularly identified key circRNAs (*CSS-circFAB1*), and experimentally revealed that *CSS-circFAB1* is likely to act as a positive regulator to mediate the cold tolerance of tea plants, although its solid functions and regulation mechanism need further examinations. It should be mentioned that the expression level of the parent gene of *CSS-circFAB1* was significantly downregulated after feeding with circRNA-specific AsODN primers. It is still unclear whether the reduced expression of the parental gene is related to the down-regulated expression of circRNAs or the low specificity of AsODN primers. The establishment of a stable genetic transformation of tea plants and the construction of parental gene mutants in the future will help to further improve the functional verification of *CSS-circFAB1*.

In conclusion, we comprehensively characterized the expression and the function of circRNAs of tea plants under chilling stress. In conjunction with mRNA and miRNA analysis and functional experiments, we provide novel insights into the sequence characteristics, expression and evolutionary patterns, and the potential roles of tea circRNAs on cold tolerance. The obtained results will not only increase our understanding of the evolution and expression dynamics of tea circRNAs, but will also underscore the crucial roles of circRNAs in regulating cold tolerance in plants.

## 4. Materials and Methods

### 4.1. Plant Materials and Chilling Treatment

The three-year-old cultivated tea plant ‘Shuchazao’ was selected and planted in pots under natural conditions at the Guohe Tea Plant Cultivar and Germplasm Resource Garden of Anhui Agricultural University (Hefei, China). Healthy and similar-growing-stage samples were moved to a growth chamber for at least one month for adaptive growing, with a 12 h/12 h day/night photoperiod at 25 °C under 70% relative humidity. Chilling stress under 4 °C were then conducted with timeline sampling at 0 h (CK), 6 h (C6), 12 h (C12), 24 h (C24), and 7 days (C7d). Tender bud or young leaves were collected at each time point and stored at −80 °C after freezing in liquid nitrogen until DNA/RNA extraction. Three biological replicates were employed for each sampling. Genomic DNA and total RNA were isolated using the modified CTAB method [43] and RNAprep Pure Plant Plus Kit (Tiangen, China) according to the manufacturer’s protocol. Qualified DNA/RNA was then used for subsequent experiments.

### 4.2. CircRNA Sequencing and Bioinformatics Analysis

Ribosomal RNA was removed from the total RNA with further RNase R treatment to digest linear RNA aiming to generate high-confidence circRNA sequences. CircRNA sequencing libraries were constructed, evaluated, and then sequenced on an Illumina Hiseq 4000 platform. SOAPnuke [44] and FastQC (https://github.com/s-andrews/FastQC, accessed on 14 October 2020) were used to trim and assess the quality of the raw and processed data. Clean reads were mapped to the tea plant reference genome [45] using BWA [46] or Bowtie2 [47]. Two pieces of high-performance software, CIRIquant [48] and Find_circ [27], were used for circRNA identification according to the manuals. The predicted circRNAs with supported back-splice junction reads greater than 2 were kept and defined as high-confidence circRNAs.

Gene length, exon length, flanking intron length, and expression level of linear genes and circRNAs were calculated based on the annotation information of the tea plant reference genome. A random sampling of linear genes to an equal number with circRNAs was adopted when comparing the characteristics of circRNAs and linear genes. Some of the scripts and parameters involved in the calculation are from https://github.com/conniecl/maize_circRNA (accessed on 22 October 2020). Flanking intron sequences of the circRNAs and the genes that do not produce circRNAs were extracted for repeat sequence annotation using RepeatMasker (https://www.repeatmasker.org/, accessed on 3 June 2021) on the basis of the Repbase [49] library and the repeat sequences of the tea plant genome. Additionally, the methylation level of circRNAs and non-circRNA genes was also investigated based on a single-base-resolution DNA methylation map constructed by whole-genome bisulfite sequencing [8].

### 4.3. Conservation Analysis of Tea Plant circRNAs

The high-confidence circRNAs were aligned against the circRNAs from 20 representative plant species in PlantcircBase (Release 7) [50] using Blastn (with E-value < 1 × 10^−5^) to investigate their conservation with other plant circRNAs in the genomic sequence level. Three strategies were adopted to evaluate the conservation of circRNAs, including (1) alignment of back-splicing junction sequences of the circRNAs between the tea plant and other plants; (2) BLAST analysis based on linear genomic sequences; (3) short sequence similarity comparisons based on the abundance of *K-mer* (https://github.com/CalabreseLab/seekr, accessed on 15 July 2021). We connected the 100 bp upstream and downstream of the circRNAs back-spliced site as the junction sequences for alignments.

### 4.4. Sequence Validation and Quantitative Real-Time PCR Assays

Twenty-five randomly selected circRNAs were employed for experimental validation. Divergent and convergent primers were designed according to the circular and linear sequences of circRNAs (Table S4). Genomic DNA and cDNA were used as PCR templates for both convergent and divergent sequence amplification. Target PCR products from the divergent primer and cDNA samples were separated using agarose gel electrophoresis and purified for Sanger sequencing, aiming to confirm the presence of back-spliced junction sites. In the quantitative real-time PCR (qRT-PCR) assay, cDNA was synthesized using the random 6-mers as primers and the SYBR Green method was used for qRT-PCR using the divergent primer (Table S16). The relative expression of each circRNA was calculated using the 2^−∆∆CT^ method. The β-actin gene in the tea plant (NCBI accession: HQ420251) was used as an internal reference control (Table S4). Three biological replicates of each PCR run were performed. 

### 4.5. Expression and Functional Enrichment Analysis

Fragments Per Kilobase of exon model per Million mapped fragments (FPKM) was applied to calculate the gene expression abundance by the RSEM package [51]. Differentially expressed genes between different treatment comparisons were characterized using EBSeq [52] with an expression fold-change of ≥2 and adjusted *p* value ≤ 0.05. The number of junction reads was normalized by the total number of clean sequencing reads (units in billions) to estimate the relative expression abundance of circRNAs, denoted as RPB (junction reads per billion clean reads) [20,20]. CircRNAs were classified into low (<100 RPB), medium (100–500 RPB), and high (>501 RPB) by their relative expression levels. Differentially expressed circRNAs between each treatment were characterized using the edgeR package [53]. The criteria for differentially expressed circRNAs were |fold-change| > 1 and *p* value ≤ 0.05. Functional enrichment analysis was conducted using the GOATOOLS package [54].

### 4.6. miRNA Identification and Prediction of Interaction Network

To investigate the candidate interactions among circRNAs, mRNAs, and miRNAs during chilling treatments in the tea plant, we first performed high-throughput sequencing of miRNA according to the preparation of small RNA libraries based on the instructions. Reads with adaptors, low-quality bases, and lengths shorter than 18 bp or greater than 41 bp were trimmed. Clean reads were then mapped to the plant miRNAs mature sequences from the miRBase database to characterize known miRNAs [55]. Based on the differential expressed circRNAs, mRNA, and miRNAs identified in the study, we constructed the circRNAs-miRNAs-mRNAs regulating network under chilling stress of the tea plant. We used psRNATarget (https://www.zhaolab.org/psRNATarget/, accessed on 8 November 2021) [56] to determine the binding relationships between circRNAs and miRNAs, and miRNAs and mRNAs according to the default parameters. Cytoscape [57] was applied to visualize the interaction network.

### 4.7. Gene Suppression and FISH Experiment

As the stable genetic transformation of tea plants has not yet been established, we adopted the antisense oligodeoxynucleotides (AsODN)-based gene silencing technology to validate the functions of tea circRNAs [13,58,59]. This method has been widely used in tea plants for gene validation [60,61]. AsODNs were identified using Soligo software (Table S17). Isolated tea plant samples with one bud and two leaves were soaked in 1 mL of 20 μM AsODN solution, and the sense oligonucleotides (sODN) were used as the control. The leaves were harvested at 0 h, 6 h, 12 h, and 24 h of incubation to test the best silencing time point, and the silencing treatment was carried out at the best silencing time followed by cold treatment of 0 °C for 1 h. Phenotypes of samples were assessed after darkness treatment for 30 min at 25 °C with no less than 3 biological replicates. The photosynthetic rate and maximum photochemical efficiency of photosystem II (*Fv/Fm*) were then measured. Meanwhile, we also determined the content of malondialdehyde (MDA) of all samples. In situ hybridization was performed using specific probes (Table S18). Probes covering the back-splice sites of circRNA were labeled with fluorescein isothiocyanate dye in the 5′ terminal, while the probe for the linear region was only labeled with trimethine cyanine dye. The FISH experiment was performed as previously described [16,62]. The sense probe for circRNA and its linear regions were designed as the control. The images were acquired by fluorescence microscopy.

### 4.8. Statistics Analysis

All experiments were performed in triplicates, and the data generated by the three experimental repeats were statistically analyzed using Microsoft-EXCEL 2010 and GraphPad Prism 8.0 (Graphpad software, http://www.graphpad.com, accessed on 20 December 2020) software. The statistical significance between groups was analyzed using Student’s *t*-test.

## Data Availability

Raw sequences of the circular RNA experiments generated in this study have been deposited into the National Center for Biotechnology Information database under BioProject accession number PRJNA893865.

## Supplementary Materials

The following supporting information can be downloaded at: https://www.mdpi.com/article/10.3390/ijms24021478/s1.
