# Supplementary material for: Evolutionary Landscape of Tea Circular RNAs and Its Contribution to Chilling Tolerance of Tea Plant
Source: Int J Mol Sci. 2023 Jan 12;24(2):1478. doi: 10.3390/ijms24021478 (PMC9861842; doi:10.3390/ijms24021478)
Supplement: Supplementary file 1 [file ijms-24-01478-s001.zip › Supplementary Figure_proofed.pdf]

Supplementary Figures

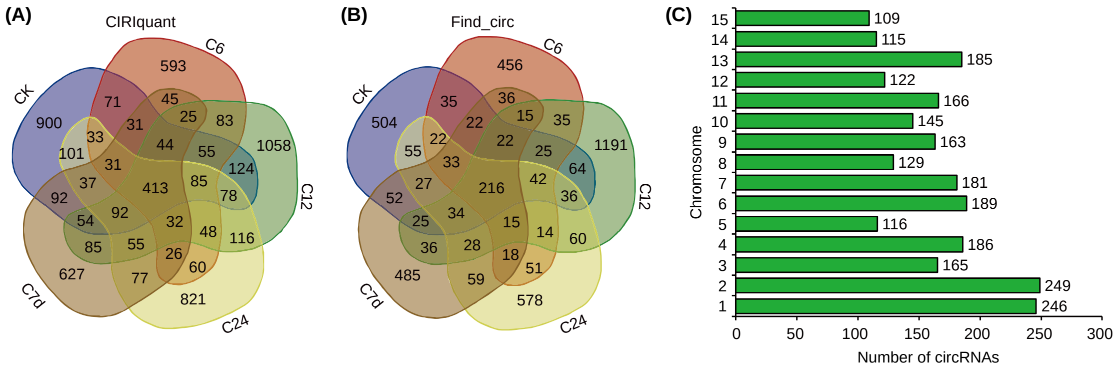

Figure S1. Identification of circular RNAs using two state-of-the-art tools and their chromosomal distribution. (A) CIRIquantand and (B) Find_circ, (C) Chromosomal distribution of circRNAs identified in tea plant.

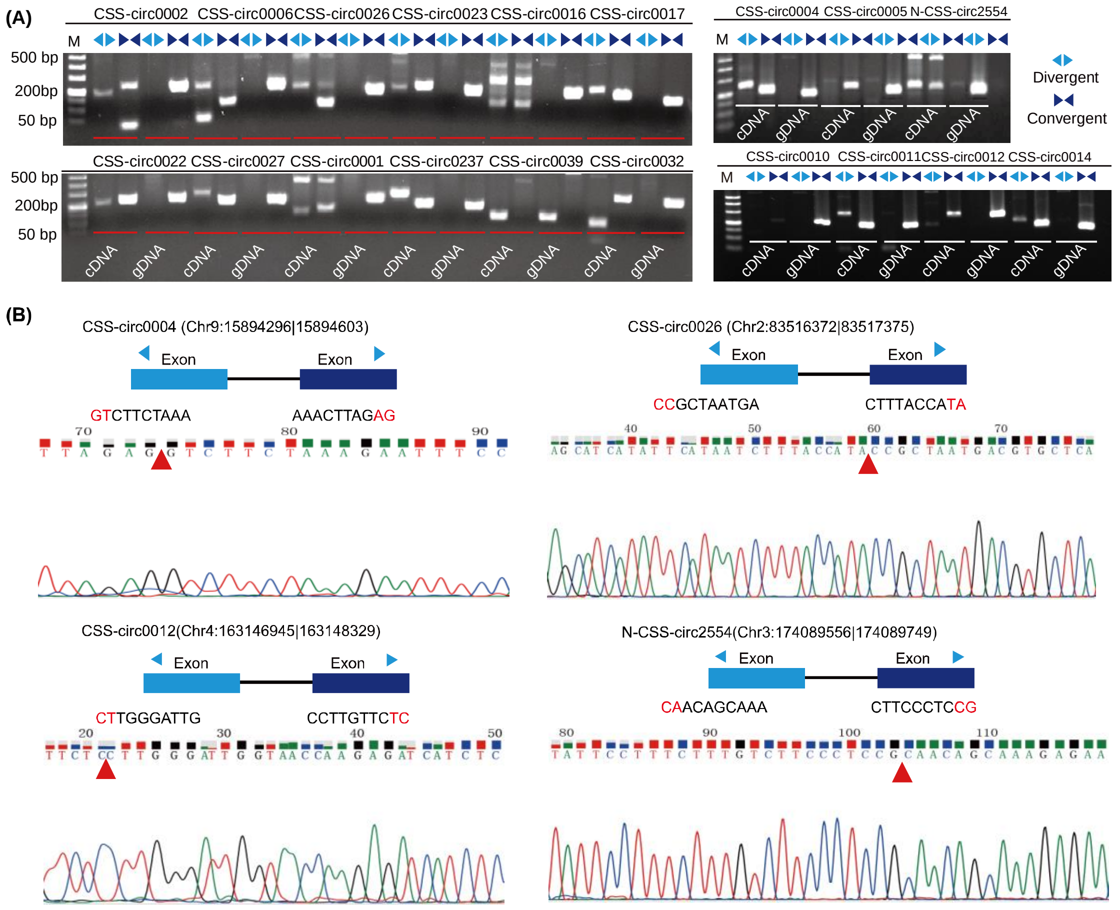

Figure S2. Sequence validation of circRNAs in tea plant. (A) PCR amplification for candidate circRNAs in genomic DNA and cDNA samples. (B) Sanger sequencing of selected circRNAs. s

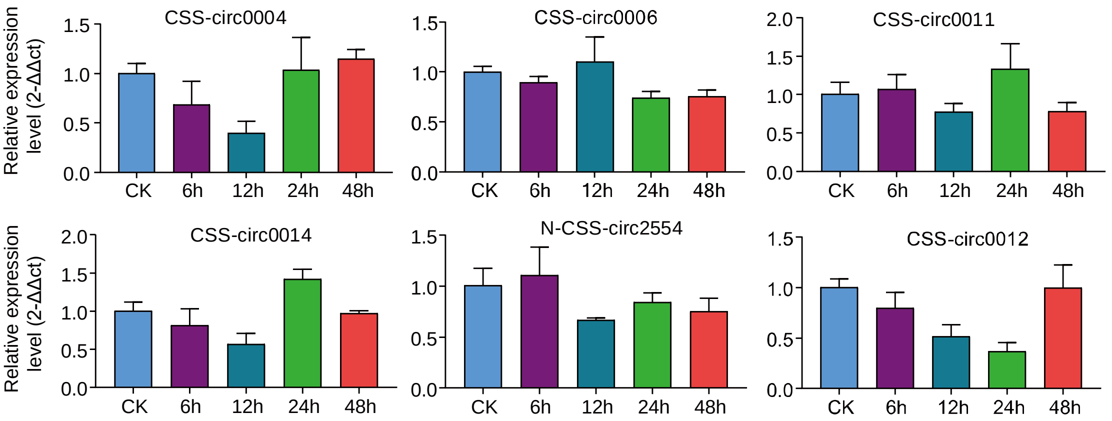

Figure S3. Expression validation of selection circRNAs in tea plant using qRT-PCR under cold treatment. $2^{-\Delta\Delta ct}$ method was used for calculating the relative expression level.

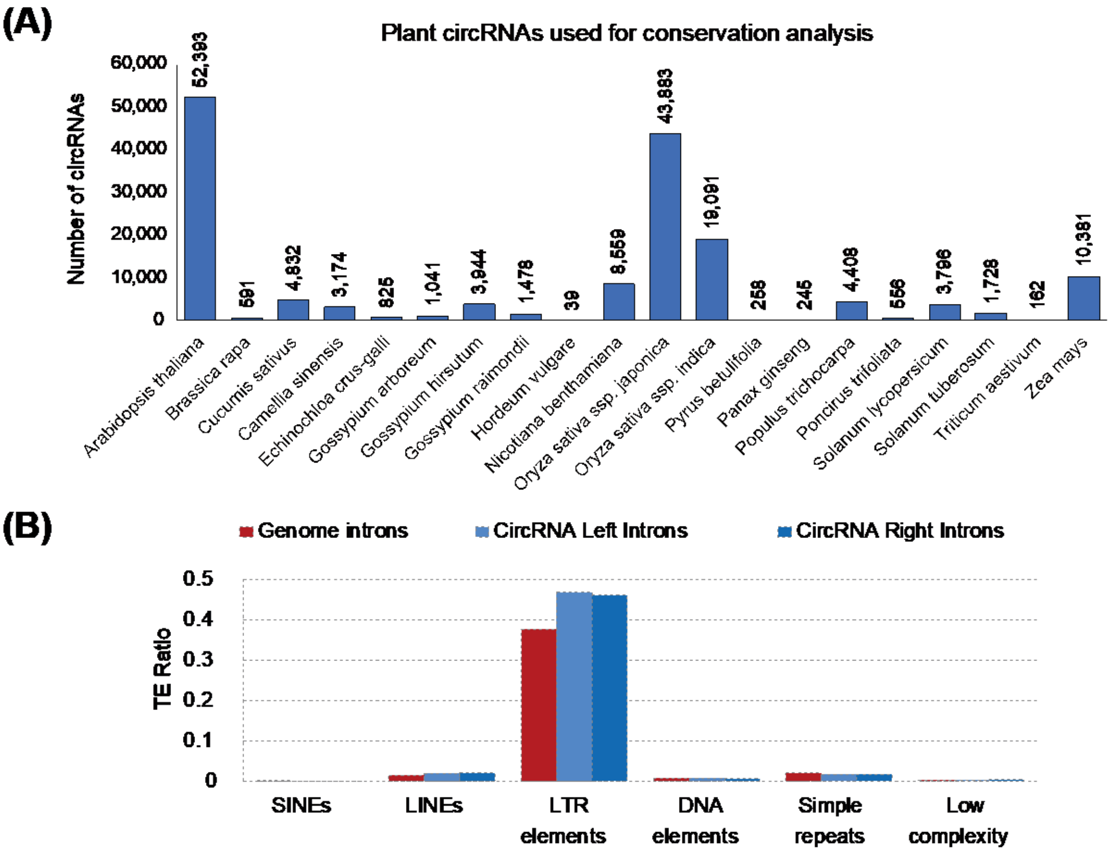

Figure S4. Conservation analysis of circRNAs. (A) Plant circRNAs in 20 representative species from PlantcircBase Release 7. (B) *K-mer* values for comparison between different species.

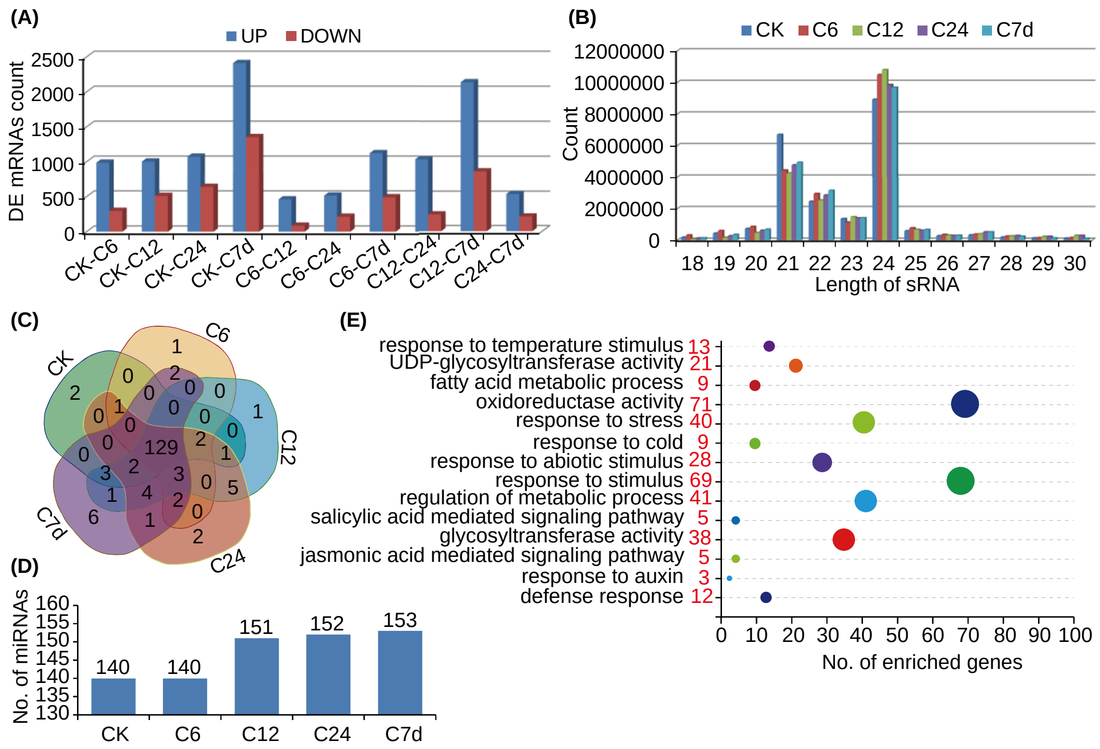

Figure S5. Number of differentially expressed mRNAs and conserved miRNAs. (A) Differentially expressed mRNAs. (B) Length distribution of small RNAs. (C) miRNAs shared among the five treatments. (D) Number of miRNAs between treatments. (E) Analysis of differential mRNAs functional enrichment in ceRNAs regulatory networks.

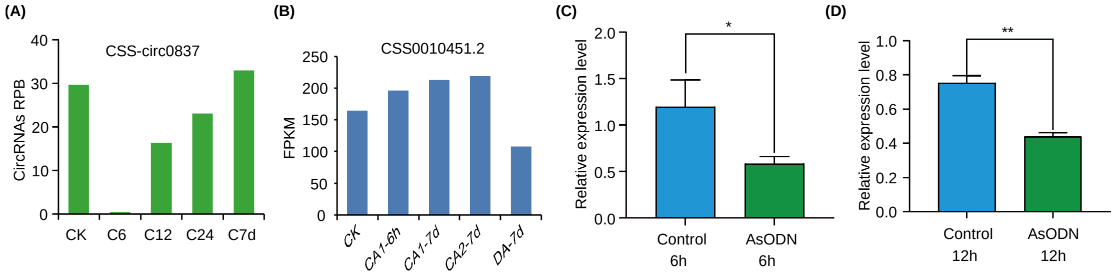

Figure S6. Expression and silencing of *CSS-circFAB1* and its parental gene *CSS0010451.2*. (A) *CSS-circFAB1* expression (RPB) under chilling treatments. (B) Expression of CSS-circFAB1 parental-gene (CSS0010451.2) under cold treatment. Expression (C, D) Expression level of *CSS-circFAB1* parental gene CSS0010451.2 for 6h and 12h after silencing *CSS-circFAB1*. * p value < 0.05; ** p value < 0.01
